# Psychological Problems in a Sample of Jordanian Healthcare Workers Involved in Caring for Patients With COVID-19: A Cross-Sectional Study

**DOI:** 10.3389/fpsyg.2021.679785

**Published:** 2021-08-26

**Authors:** Fadwa Naji Alhalaiqa, Anas H. Khalifeh, Omar Al Omari, Dalal Bashier Yehia, Malek Mohammad Hassan Khalil

**Affiliations:** ^1^Psychiatric Mental Health, Faculty of Nursing, Philadelphia University, Amman, Jordan; ^2^Department of Nursing, Hittien College, Amman, Jordan; ^3^Department of Nursing, Prince Hamzah Hospital, Ministry of Health, Amman, Jordan; ^4^Collage of Nursing, Sultan Qaboos University, Muscat, Oman; ^5^Faculty of Nursing, Al-Zaytoonah University of Jordan, Amman, Jordan; ^6^Faculty of Nursing/Psychiatric Mental Health Nursing, Zarqa Private University, Zarqa, Jordan

**Keywords:** anxiety, COVID-19, depression, stress, resilience

## Abstract

**Purpose:** To explore the psychological problems (stress, depression, and anxiety) and the level of resilience among frontline healthcare workers (HCWs) who provide care for patients with coronavirus disease 2019 (COVID-19). Additionally, the current study identified the correlation among these problems, resilience, and demographics of participants. The study explored the main predictors of stress, depression, anxiety, and resilience.

**Methods:** Both the descriptive cross-sectional correlational design and convenience sample technique were used to collect data from active Jordanian HCWs who directly deal with patients suspected or confirmed to be infected with COVID-19 and work at healthcare facilities in Jordan. Data were collected by using online questionnaires about the demographics, stress, anxiety, depression, and resilience of the participants.

**Results:** Data were collected from 225 HCWs. Their mean age was 31.17 years (SD = 6.8). All the participants perceived different levels of stress, with most perceiving exposure to a high level of stress (distress) (46.2% with low level and 53.8% with high level of stress); approximately half of them (52.9%; *n* = 119) reported a high level of anxiety, and more than half (66.2%; *n* = 149) had a high level of depression. Additionally, an increased anxiety and depression level was significantly associated with the decreased resilience and increased stress level. Increased age and experience of HCWs are significantly correlated with increased stress levels. The participants having personal protection equipment (PPE) reduced anxiety and depression and increased resilience (*p* > 0.05). The predictors of the main variables were varied in the study.

**Conclusion:** Frontline HCWs involved in treating the patients with COVID-19 are liable to have a high level of stress that is associated with increased anxiety and depression levels. These high levels affect their mental well-being and resilience. Healthcare institutions in Jordan must tailor appropriate psychological interventions and support that are congruent with the needs of HCWs during and after caring for patients with COVID-19.

## Introduction

The coronavirus disease 2019 (COVID-19) pandemic has increased the global health burden in terms of morbidity, mortality, and health expenditure (World Health Organization, 2020). Therefore, many countries exerted huge efforts to stop the spread of the virus (e.g., a quarantine, lockdown, travel restrictions, social distancing, and wearing face masks) (World Health Organization, 2020). Similar efforts were applied in Jordan (e.g., closing of schools and universities, cancellation of all public events and gatherings, and stopping prayer in all the mosques and churches) as a precaution and preventive measure.

The number of new cases in Jordan has fluctuated and has not been stable from March 2020 to the time of this writing in April 2021. To face the COVID-19 pandemic, some hospitals were assigned to deal only with confirmed cases of COVID-19; therefore, healthcare workers (HCWs) received special training to provide appropriate healthcare services to these cases (Roya, 2020). New field hospitals were also built (Prime Ministry of Jordan, 2020). Personal protection equipment (PPE), staffing, beds, and mechanical ventilator devices were provided (Prime Ministry of Jordan, 2020). As a result, the workload and stress of the frontline HCWs who dealt with patients with COVID-19 increased, particularly with the growing number of daily new and death cases and hearing news about new mutated chains of the COVID-19 virus and rumors related to the effectiveness of the vaccine (Jordan Armed Forces, 2020). Consequently, their physical and psychological health status was negatively affected (Wu et al., 2009; Kang et al., 2020).

### Background

Stress and anxiety are psychological problems. Stress is a feeling of tension and pressure in response to external factors. Meanwhile, anxiety is the internal perception and response in which a high level of this perception is associated with many disorders (e.g., post-traumatic stress disorder) (Selye, 1975; McLaughlin and Hatzenbuehler, 2009).

The sudden outbreak of public health events always associates with psychological health problems and poses major challenges to the mental health services system, as seen in previous worldwide pandemics [e.g., severe acute respiratory syndrome (SARS) in 2002 and 2003, the Middle East respiratory syndrome coronavirus (MERS-CoV) in 2012, and the Zika virus outbreak in 2016] (Morens and Fauci, 2013; Bloom and Cadarette, 2019). However, the prevalence rate of emotional exhaustion, depersonalization, and low personal accomplishment was similar to the figure recorded for HCWs exposed to chronic occupational stress and poor work organization during non-epidemic periods (Magnavita et al., 2021). Meanwhile, a review of 11 studies investigating the psychological well-being of HCWs during COVID-19 found that the reported prevalence of anxiety, depression, and stress among HCWs was very high (67.55, 55.89, and 62.99%, respectively) (Vizheh et al., 2020).

Currently, the ongoing COVID-19 pandemic stress is causing many psychological problems for all people (e.g., fear, anxiety, distress, and depression) (Duan and Zhu, 2020; Mekonen et al., 2020; Pappa et al., 2020; Xiang et al., 2020). A study conducted by Magnavita et al. (2020) assessed the symptoms among HCWs during COVID-19. They found that 16.6 and 20.3% of participants reported anxiety and depression, respectively. However, these prevalence were similar to the results during periodic checks. Dai et al. (2020) conducted a descriptive cross-sectional study on 4,357 HCWs in China dealing with confirmed and suspected cases of COVID-19, especially in Wuhan. They reported that 39.1% of HCWs had psychological distress; all of them were worried and felt fear and distress because they were the first-line treatment providers for confirmed cases of COVID-19. Moreover, those HCWs were afraid of infecting their families, colleagues, and other loved ones. Therefore, a timely understanding of mental health issues is urgently needed not only for society but also for HCWs (Xiang et al., 2020).

In the face of this widespread infectious public health event, HCWs are under severe physical and psychological stress (Wu et al., 2009; Kang et al., 2020; Spoorthy et al., 2020; Guo et al., 2021). The frontline HCWs, such as nurses and physicians, are the most vulnerable people to have emotional instability because they are dealing with confirmed and suspected cases of COVID-19 (Lima et al., 2020; Spoorthy et al., 2020). HCWs in hospitals suffer from psychological problems, such as anxiety, distress, and depression as a result of many causes [e.g., feeling stigmatized, fear of infecting themselves or others/their families, feeling isolated, and eating alone (since they are in close contact with infected patients)], working for long hours to cover all shifts in the hospitals in these conditions (Maunder et al., 2003; Pappa et al., 2020; Spoorthy et al., 2020; Zandifar and Badrfam, 2020). The psychological problems of frontline HCWs are correlated with maladaptive coping (Maunder et al., 2003; Pappa et al., 2020; Spoorthy et al., 2020).

In the Arabic region, a few studies were conducted in the Gulf area. In Saudi Arabia, the psychological impact of COVID-19 was explored using the 9-item Patient Health Questionnaire (PHQ-9) to measure depression and the 7-item Generalized Anxiety Disorder Scale (GAD-7) to measure anxiety. It was found that a large number of participants experienced normal to mild depression and minimal to mild anxiety. However, findings were limited by including all HCWs, regardless of whether they were dealing with patients with COVID-19 (Al Ammari et al., 2021). In the Sultanate of Oman, 150 frontline HCWs experienced a high level of distress [measured by the Perceived Stress Scale 10 (PSS-10)] and anxiety (measured by the GAD-7) (Al Mahyijari et al., 2020). Working with patients with COVID-19 negatively affects not only the mental well-being of HCWs but also their resilience (Conversano et al., 2020; Kalaitzaki et al., 2020; Santarone et al., 2020; Siyu et al., 2020; Zhu et al., 2020). Resilience is defined as “involving positive adaptation to adversity” (Robertson et al., 2016). It is positively associated with mental health (Li et al., 2021). The resilience of HCWs is affected by personal characteristics (e.g., gender, alcohol abuse, personality features), physical and mental health, and a protective work environment (e.g., availability of resources) (Robertson et al., 2016). Age was correlated with resilience, where adults and older adults experienced higher resilience than the young (Li et al., 2021). A review study conducted by Conversano et al. (2020) found that the resilience factors of healthcare professionals involved in COVID-19 are effective social support, self-efficacy, locus of control, and environmental factors. The resilience of nurses and HCWs was reported as an essential direct predictor of their mental health (Park et al., 2018). Also, more healthcare experience enhances the resilience of HCWs who will face future disease outbreaks (Maunder et al., 2006).

The sociodemographic characteristics of HCWs (e.g., gender, age, profession/occupational differences, place of work, and psychological variables, such as poor social support and self-efficacy) are associated with increased prevalence and severity of mental health problems (e.g., anxiety, depression, and distress) (Pappa et al., 2020; Spoorthy et al., 2020). Women and nurses were more liable to have anxiety and depression than men and doctors, respectively (Du et al., 2020; Pappa et al., 2020). It is worth noting that despite the common mental disorders and problems that exist among HCWs in such sites, most healthcare professionals working in isolation units and hospitals do not receive any personal or patient mental healthcare training programs (Siyu et al., 2020; Xiang et al., 2020; Zhu et al., 2020). Different methods were used to measure distress, depression, and anxiety among healthcare providers during COVID-19: PSS-10; Hopkins Symptom Checklist (HSCL-25); GAD-7; PHQ-9; and Depression, Anxiety, and Stress Scale-21 (DASS) (Babore et al., 2020; Ma et al., 2020; Wilson et al., 2020). Therefore, it is crucial to identify the psychological impact and associated factors of HCWs caring for patients with COVID-19 to develop a plan for psychological aids that help HCWs decrease the harmful psychological effect of this extraordinary pandemic.

Few studies have explored the psychological impact of the COVID-19 outbreak among HCWs in the Arabic region. Most studies have been conducted in the Gulf area. To the best of our knowledge, few studies have been conducted in Jordan to assess the mental health impact and associated factors among HCWs dealing with patients with COVID-19. The first study focused on acute stress disorders and predictors of psychological distress (Shahrour and Dardas's, 2020). The second one examined the level of stress, anxiety, and depression using the DASS. The study found that HCWs experienced depression, anxiety, and stress (40, 60, and 35%, respectively) (Alnazly et al., 2021). In the current study, the term distress is used to indicate the damage suffered by HCWs. To focus on sociocultural context; therefore, the purpose of the current study is to explore the psychological problems (distress, anxiety, and depression) and to examine the relationship between the level of resilience and mental health problems among frontline HCWs who provide care for patients with COVID-19. Most prior studies did not examine the predictors of distress, anxiety, depression, and resilience; in the current study, we aimed to explore these predictors.

## Materials and Methods

### Design

A descriptive cross-sectional correlational design was used to meet the purposes of the current study. The data were collected from participants *via* online questionnaires in March–July 2020. The strengthening of the reporting of the observational studies in epidemiology (STROBE) guidelines was used in reporting.

### Sample and Setting

A convenience sample technique of Jordanian HCWs was used. In the present study, active Jordanian HCWs directly dealing with patients suspected or confirmed to be infected with COVID-19 and working at healthcare facilities in Jordan were included. The exclusion criteria were HCWs who were previously or currently infected (there were four) and those who did not deal with patients with COVID-19 (there were 10). The sample size was determined by using G^*^ power 3.1.7 software (Faul et al., 2013). In the study, the correlation test was used for estimating the sample size (using power = 0.95, alpha = 0.05 two-tailed, effect size = 0.3), therefore, the minimum sample size was calculated to be 138. To accommodate refusals and dropouts, a convenience sample of 200 HCWs was expected.

### Ethical Considerations

Ethical approval was obtained from the scientific research committee institutional review board of Philadelphia University. The rights of participants (e.g., withdrawal, confidentiality, and privacy) were maintained. An online consent form was obtained from all participants.

### Data Collection Procedure

The researchers used the network approach to recruit a sample for the study from different hospitals (public, private, educational, and Royal Medical Services). The researchers also used an online network to announce the study; this was done by sending the announcement through the social media accounts of the researchers (e.g., WhatsApp, Facebook). The announcement was an information sheet that explained the purpose, procedure, possible risks, side effects, and discomfort of the research study and the rights of participants, and it provided contact information for the researchers. Those who expressed interest in participating contacted the researchers, who were sent the electronic questionnaire (Google Forms) through WhatsApp. All the participants who contacted the researchers completed the questionnaire. The first page of the electronic questionnaire contained a consent form; the rest of the pages were the questionnaire. A single response to the questions was permitted for each participant. Each participant was assigned an ID code to protect his or her identity. The survey questionnaire required >20 min to complete. All data were kept on a designated hard disk at the office of the principal researcher.

### Instruments

Four instruments were used in this study. We used the PSS-10, since it reflects the individual perception of distress that indicates the damage suffered by HCWs, and the HSCL-25, because it measures the presence and intensity of depression and anxiety (Hesbacher et al., 1980). Meanwhile, the Resilience Scale for Nurses (RSN-19) was used to measure the ability required by nurses to overcome the challenges that they meet in the clinical setting (Park et al., 2019) (refer the supplementary file for further details regarding the questionnaires):

#### Demographic and Clinical Questionnaire

This questionnaire was designed by the current researcher and gathered information with two levels of measurement such as continuous (e.g., age and years of experience) and categorical (e.g., gender and marital status) (Table 1 presents all items gathered through this questionnaire).

**Table 1:** Characteristics of the participants (*n = 225).

**Variables**	**Freq**.	**%**
**Age**	(M = 31.17, SD = 6.8)	
**Years of experience**	(Media *n* = 7.00)	
**Gender**		
Male	95	42.2
Female	130	57.8
**Marital status**		
Single	91	40.4
Married	123	54.7
Divorced	11	4.9
**Do you live alone?**		
Yes	17	7.6
No	208	92.4
**Specialty**		
Doctor	15	6.7
Nurse	192	85.3
Other (pharmacist, laboratory technician, radiologist, nutritionist)	18	8.1
**Degree**		
Bachelor's degree	172	76.4
Postgraduate studies (higher diploma, master's, and doctoral degree)	53	23.6
**Type of hospital working in (in terms of the owner of the hospital)**		
Public	114	50.7
Private	78	34.7
Educational	17	7.6
Royal Medical Services	16	7.1
**Patients worked with regarding COVID-19**		
Suspected	182	80.9
Confirmed	37	16.4
Both	6	2.7
**Working hours during COVID-19**		
A, B, C	108	48.0
Day/night	85	37.8
Full day	32	14.2
**Does your hospital or department have adequate personal protection equipment?**		
Yes	173	76.9
No	52	23.1
**Stress level**		
Low level	104	46.2
High level	121	53.8
**Anxiety level**		
No anxiety	106	47.1
High level of anxiety	119	52.9
**Depression level**		
No depression	76	33.8
High level of depression	149	66.2

*N, number of participants*.

#### PSS-10

This instrument was developed by Cohen (1988). The scale is a self-reported questionnaire developed to measure the perception of distress and to determine how unpredictable, uncontrollable, and overloaded respondents find their lives (Cohen, 1988). It is a 5-point Likert scale ranging from 0 = never to 4 = very often. Total scores range from 0 to 40. Scores of 20 or higher are considered a high level of distress.

In the Arabic version of the PSS-10, Cronbach's alpha was good for both paper and electronic form (α = 0.72 and α = 0.85, respectively) (Ben Loubir et al., 2014). In the current study, Cronbach's alpha was good (α = 0.82).

#### HSCL-25

This is a self-report questionnaire developed to measure the presence and intensity of depression and anxiety symptoms, derived from the 90-item Symptom Checklist (SCL-90) (Derogatis et al., 1974). It includes two sections: the first section includes 10 items about anxiety, and the second section includes 15 items about depression (Hesbacher et al., 1980). It uses a 4-point Likert scale (1 = not at all, 2 = a little, 3 = quite a bit, and 4 = extremely) (Hesbacher et al., 1980). The patients who scored greater than or equal to 1.75 had depression or anxiety (Winokur et al., 1984).

The Arabic version assessed women in the general population and showed good internal consistency. Cronbach's alpha was 0.92 for all scales and 0.88 and 0.85 for depression and anxiety subscales, respectively (Al-Turkait and Ohaeri, 2010; Mahfoud et al., 2013). Meanwhile, in the current study, Cronbach's alpha was 0.90 for all scales and 0.86 and 0.83 for depression and anxiety subscales, respectively.

#### RSN-19

This scale was developed to measure the ability required by nurses to positively overcome many difficulties in clinical settings (Park et al., 2019). The RSN-19 comprised 19 items. Each item was scored using a 5-point Likert scale, ranging from 1 (almost never) to 5 (almost always), with higher scores indicating a greater degree of resilience. In the current study, this instrument was translated into Arabic according to the WHO guideline. The pilot study with 10 participants showed good reliability for the whole scale (Cronbach's alpha of 0.884) and subscales (Cronbach's alpha of 0.813 for the philosophical pattern, 0.706 for the relational pattern, 0.777 for the situational pattern, and 0.777 for the dispositional pattern).

### Data Analysis Plan

The data were downloaded in an Excel form and exported to the SPSS program version 26. The sample characteristics were described using mean, SD, frequencies, and percentages. An independent *t*-test was used to explore whether the total mean score for the PSS-10, RSN-19, HSCL Anxiety, and HSCL Depression was different based on gender and the availability of personal protection. An ANOVA test was conducted to determine the differences between the total mean score for the dependent variables of the PSS-10, RSN-19, HSCL Anxiety, and HSCL Depression, and the independent variables (stress that causes distress) of marital status, the place of living, specialty, qualification of the participants, type of hospital, the nature of the patients whom participants have dealt with regarding COVID-19, and the nature of the shift the participants work. Pearson's correlation was used to explore the relationship among the PSS-10, RSN-19, HSCL Anxiety, HSCL Depression, age, and years of experience. A regression analysis was performed to determine the main predictors of resilience, stress, anxiety, and depression. The data were analyzed at a significance level of *p* < 0.05 and two-tailed to generate descriptive and inferential statistics.

## Results

### Demographic and Clinical Characteristics

There were no missing data; therefore, 225 participants took part in this study, with a mean age of 31.17 years (SD = 6.8) and 8.21 years of experience (SD = 6.497). The majority of participants were women (130; 57.8%), married (123; 54.7%), and do not live alone (208; 92.4%). Two-thirds of the participants (172; 76.4%) hold a bachelor degree, and half of the participants work in a public hospital (as shown in Table 1).

### Prevalence of Psychological Problems

Table 1 shows that all participants have stress. In total 46.2% (*n* = 104) of the participants perceived a low level of stress, while 53.8% (*n* = 121) perceived exposure to high-level distress. Regarding the anxiety level, the results showed that approximately half of the study participants (52.9%; *n* = 119) reported a high level of anxiety, and more than half of them (66.2%; *n* = 149) had a high level of depression.

### Differences Between Psychological Problems and Some Demographic and Clinical Characteristics

An ANOVA test was conducted to determine the differences between the total score mean for the independent variables of the PSS-10, RSN-19, HSCL Anxiety, and HSCL Depression and the dependent variables of marital status, the place of living, specialty, qualification of the participants, type of hospital, the nature of the patients whom participants have dealt with regarding COVID-19, and the nature of the shift in which the participants work. There was no significant difference between the means except for speciality in relation to stress (PSS-10) [*F*_(6.2018)_ = 2.688, *P* = 0.015] and type of patients (confirmed or suspected case) in regards to the HSCL Depression [*F*_(2.222)_ = 3.696, *P* = 0.026].

### Differences in Psychological Problems in Terms of Gender and Having PPE

As shown in Table 2, an independent samples *t*-test was conducted to compare the PSS-10, RSN-19, HSCL Anxiety, and HSCL Depression in regard to gender. There was a significant difference in the anxiety score for men (M = 17.14, SD = 4.6) and women [M = 20.26, SD = 5.39; *t*_(210)_ = −4.44, *p* < 0.001]. There was also a significant difference in the depression score between men (M = 28.17, SD = 8.2) and women [M = 32.46, SD = 9.17; *t*_(223)_ = −3.621, *p* < 0.001] (as shown in Table 2).

**Table 2:** Independent *t*-test results of the PSS, Resilience Scale for Nurses (RSN), HSCLA, HSCLD, and gender.

	**Gender, M (SD)**				
**Variable**	**Male**	**Female**	**t**	**df**	***p***
^*^PSS	22.04 (4.46)	22.34 (4.45)	−0.506	223	0.613
Resilience	73.13 (11.19)	73.1 (11)	0.025	223	0.980
^*^HSCLA	17.14 (4.6)	20.26 (5.39)	−4.442	210	0.000
^*^HSCLD	28.17 (8.2)	32.46 (9.17)	−3.621	223	0.000

* *PSS, Perceived Stress Scale*.

* *HSCLA, Hopkins Symptom Checklist for Anxiety*.

* *HSCLD, Hopkins Symptom Checklist for Depression*.

An independent *t*-test was also conducted to compare the means of the PSS-10, RSN-19, HSCL Anxiety, and HSCL Depression in regard to the availability of adequate PPE. There was a significant difference in anxiety and depression, with significantly higher scores in the groups that do not have adequate PPE, and their resilience was significantly lower compared with their counterparts who have adequate PPE (*p* < 0.001) (as shown in Table 3).

**Table 3:** Independent *t*-test results of the PSS^*^, RSN^*^, HSCLA^*^, HSCLD^*^, and personal protection equipment (PPE*).

	**PPE, M (SD)**				
**Variable**	**Yes**	**No**	**t**	**df**	***p*-value**
PSS	22.2 (4.52)	22.4 (4.23)	−0.308	223	0.758
Resilience	75.1 (10.9)	66.7 (8.90)	4.997	223	0.000
HSCLA	18.3 (4.96)	21.2 (5.86)	−3.387	210	0.001
HSCLD	29.3 (8.69)	35.2 (8.64)	−4.277	223	0.000

* *PSS, Perceived Stress Scale*.

* *RSN, Resilience Scale for Nurses*.

* *HSCLA, Hopkins Symptom Checklist for Anxiety*.

* *HSCLD, Hopkins Symptom Checklist for Depression*.

### Correlation Between Psychological Problems and Specific Participant Characteristics

Pearson's correlation analysis showed a significant and positive correlation among the PSS-10, HSCL Anxiety, HSCL Depression, age, and years of experience. A reverse significant correlation was found among resilience, anxiety, and depression. When anxiety and/or depression increased, the resilience among health team members decreased (as shown in Table 4).

**Table 4:** Correlations among the PSS-10, RSN-19, HSCL Anxiety, HSCL Depression, age, and years of experience.

	**1**	**2**	**3**	**4**	**5**	**6**
1. ^*^PSS	–	0.056	0.346^**^	0.348^**^	0.153^*^	0.144^*^
2. RSN	0.056	–	−0.278^**^	−0.416^**^	−0.060	0.028
3. ^*^HSCLA	0.346^**^	−0.278^**^	–	0.767^**^	−0.007	−0.068
4. ^*^HSCLD	0.348^**^	0.416^**^	0.767^**^	–	0.036	−0.016
5. Age	0.153^*^	−0.060	−0.007	0.036	–	0.897^**^
6. Years of experience	0.144^*^	0.028	−0.068	−0.016	0.897^**^	–

* *Correlation is significant at the 0.05 level (two-tailed)*.

** *Correlation is significant at the 0.01 level (two-tailed)*.

* *PSS, Perceived Stress Scale*.

*RSN, Resilience Scale for Nurses*.

* *HSCLA, Hopkins Symptom Checklist for Anxiety*.

* *HSCLD, Hopkins Symptom Checklist for Depression*.

### Predictors of Resilience, Stress, Anxiety, and Depression

The regression analysis was conducted to determine the predictors of the main variables: resilience, stress, anxiety, and depression. The results showed the following:

#### RSN-19

The final model was statistically significant compared to the constant (*F* = 5.388, *p* < 0.001), with final variables of age, years of experience, and having adequate PPE as significant predictors of resilience. The values of *R*^2^ and adjusted *R*^2^ of the final model were 0.131 and 0.106, respectively (Table 5, model 1).

**Table 5:** Predictors of resilience, stress, anxiety, and depression.

**Model one: predictors of resilience**	**Unstandardized coefficients**		**Standardized coefficients**			**95.0% confidence interval for B**	
	**B**	**Std. error**	**Beta**	**t**	**Sig**.	**Lower bound**	**Upper bound**
(Constant)	95.954	8.999		10.662	0.000	78.216	113.692
Age of participants	−0.630	0.239	−0.385	−2.641	0.009	−1.101	−0.160
Live alone with reference to yes	1.958	2.687	0.047	0.729	0.467	−3.338	7.255
How many years of experience?	0.596	0.247	0.349	2.416	0.017	0.110	1.083
Personal protection equipment with reference to yes	−8.059	1.685	−0.308	−4.784	0.000	−11.380	−4.739
Marital status with reference to single	−0.893	1.511	−0.040	−0.591	0.555	−3.870	2.085
Working with actual COVID-19 patients with reference to suspected	−0.484	1.859	−0.017	−0.261	0.795	−4.148	3.179
**Model two: predictors of** ***stress***							
(Constant)	16.218	5.411		2.997	0.003	5.552	26.884
Age of participants	0.403	0.137	0.430	2.940	0.004	0.133	0.673
Live alone with reference to yes	−1.661	1.531	−0.070	−1.085	0.279	−4.678	1.357
How many years of experience?	−0.465	0.141	−0.476	−3.292	0.001	−0.743	−0.187
Marital status with reference to single	2.740	0.874	0.214	3.134	0.002	1.017	4.463
The nature of your hours during the time of the presence of the coronavirus	−1.557	0.880	−0.123	−1.768	0.078	−3.292	0.179
Private with reference to public	−2.274	1.032	−0.171	−2.204	0.029	−4.307	−0.240
Working with actual COVID-19 patients with reference to suspected	−2.558	1.092	−0.160	−2.343	0.020	−4.711	−0.406
**Model three: predictors of** ***anxiety***							
1 (Constant)	1.865	0.485		3.845	0.000	0.909	2.821
Age of participants	0.022	0.012	0.266	1.807	0.072	−0.002	0.047
Do you live alone?	−0.241	0.137	−0.113	−1.767	0.079	−0.510	0.028
How many years of experience?	−0.026	0.013	−0.297	−2.042	0.042	−0.051	−0.001
Marital status with reference to single	0.356	0.078	0.310	4.573	0.000	0.203	0.510
Private with reference to public	−0.186	0.092	−0.155	−2.014	0.045	−0.368	−0.004
Working with actual COVID-19 patients with reference to suspected	−0.212	0.098	−0.148	−2.160	0.032	−0.406	−0.019
**Model four: predictors of** ***depression***							
(Constant)	2.187	0.511		4.277	0.000	1.179	3.194
Age of participants	0.027	0.013	0.305	2.072	0.039	0.001	0.053
Do you live alone with reference to years	−0.322	0.144	−0.143	−2.235	0.026	−0.605	−0.038
How many years of experience?	−0.026	0.013	−0.276	−1.901	0.059	−0.052	0.001
Marital status with reference to single	0.322	0.082	0.265	3.917	0.000	0.160	0.484
The nature of your hours during the time of the presence of the coronavirus	−0.145	0.085	−0.121	−1.707	0.089	−0.312	0.022
Private hospital with reference to public	−0.211	0.097	−0.167	−2.170	0.031	−0.403	−0.019
Working with actual COVID-19 patients with reference to suspected	−0.309	0.104	−0.204	−2.988	0.003	−0.513	−0.105

#### Stress

The final model was statistically significant compared to the constant (*F* = 5.047, *p* < 0.001), with final variables of age, years of experience, being married, working in a private hospital, and working with suspected cases as significant predictors of stress. The values of *R*^2^ and adjusted *R*^2^ of the final model were 0.142 and 0.114, respectively (Table 5, model 2).

#### Anxiety

The final model was statistically significant compared to the constant (*F* = 4.938, *p* < 0.001). Significant predictors of anxiety included being married, working in a private hospital, and working with suspected cases. *R*^2^ and adjusted *R*^2^ of the final model were 0.100 and 0.096, respectively (Table 5, model 3).

#### Depression

The final model was statistically significant compared to the constant (*F* = 5.029, *p* < 0.001). Significant predictors of anxiety, such as age, living alone, being married, working in a private hospital, and working with suspected cases. *R*^2^ and adjusted *R*^2^ of the final model were 0.159 and 0.127, respectively (Table 5, model 4).

## Discussion

The results of the current study showed that more than half of HCWs (who provided care for patients suspected of or infected with COVID-19) had a high level of stress, anxiety, and depression. The stress level was different in terms of the specialty of HCWs. Meanwhile, the depression level was different in terms of the type of patients. Additionally, increased anxiety and depression levels were associated with decreased resilience and increased stress levels. Increased age and experience of HCWs are correlated with increased stress levels. Having adequate PPE reduced anxiety and depression and increased the resilience of the participants. In addition, 23% of the participants reported that they did not have adequate PPE, which may play a role in increasing the stress, anxiety, and depression of the participants.

A systematic review was conducted by Pappa et al. (2020) to determine the prevalence of depression, anxiety, and insomnia among HCWs during the COVID-19 outbreak. Their findings revealed that the prevalence of anxiety was 23.2% and depression was 22.8%. This is consistent with the findings of the current study in that HCWs had anxiety and depression. However, the current study prevalence rate is higher (more than 50% of participants). This could be rationalized by the study findings in which the main predictors of depression were age, living alone, and being married. Alnazly et al. (2021) found that the 60% of HCWs in Jordan who participated in their study experienced anxiety; however, they did not determine the type of setting of the participants. These differences might be rationalized by the use of different instruments (DASS) and differences in the demographic characteristics of participants. Gender and occupational differences (being women and a nurse) exhibit higher rates of anxiety, depression, and stress (Lai et al., 2020; Pappa et al., 2020; Al Ammari et al., 2021). The current study findings support this result. However, being men and being married were associated with a high level of depression, stress, and anxiety among Jordanian HCWs in the study by Alnazly et al. (2021). These differences between findings show the need to conduct future research with a large sample size focusing on the correlation of demographics with these psychological distresses. In the current study, the findings highlighted that the main predictors of anxiety are being married, working in private hospitals, and working with suspected cases. These could be explained by fearing being a source of infection to their kids and family.

The frontline HCWs who are treating patients with COVID-19 had a high-stress level (71%), which increased anxiety and depression (Li et al., 2021). This is consistent with the current study findings. However, the findings revealed that all participants had stress, which might have resulted from feelings of vulnerability, concerns about personal and family health, supply shortage (e.g., PPE), lack of willingness of people to be vaccinated in Jordan, and the presence of new mutated strains of the virus characterized by rapid spreading among all age groups and high fatality. Additionally, some demographic characteristics (e.g., being a woman and a nurse) might also explain the findings of the present study. Therefore, frontline HWCs, particularly women and nurses, need intense mental health interventions. The current study findings support the results of Shahrour and Dardas (2020) study, in which more than 41% of Jordanian nurses were suffering from acute stress disorders.

In a cross-sectional comparative study, younger Jordanian nurses were suffering more from psychological distress than older ones (Shahrour and Dardas's, 2020). However, the present study findings showed that increased HCW age correlated with an increased stress level, which is consistent with the studies of Alnazly et al. (2021) and Cai et al. (2020). This could be rationalized by including all healthcare providers, not only nurses, in the current study. However, this correlation is not consistent, since a negative correlation between age and stress was found by Kushal et al. (2018). This could be explained by the fact that HCWs involved with COVID-19 are exhausted because of prolonged work hours, personal concerns, family health, and self-perception of lack of PPE. This highlights the current study findings in which the main predictors of stress were age, years of experience, and having adequate PPE. Kushal et al. (2018) reported a negative correlation between the length of experience and stress. This might be because HCWs had been exposed to many challenges and stressors and learned appropriate skills. Meanwhile, the findings of the current study suggest that increased length of experience is linked to a high level of stress. The current study findings are consistent with Alnazly et al. (2021) and could be explained by the nature of the virus (which is easily spread and easily mutated for new strains), self-perception of a lack of PPE, and worldwide coronavirus news and precautions. Therefore, future studies must explore the correlation among stress, age, and experience over a prolonged period with a longitudinal design and with the availability of PPE.

The resilience of HCWs who are caring for patients with COVID-19 is affected negatively when they suffer mental health problems (e.g., stress, anxiety, and depression) (Conversano et al., 2020; Kalaitzaki et al., 2020; Santarone et al., 2020). Additionally, a shortage of PPE affects the resilience of HCWs and their ability to cope with the pandemic (Kalaitzaki et al., 2020). These results are congruent with the findings of the current study, in which the resilience of participants was reduced by increased anxiety, depression, and having adequate PPE. Furthermore, the findings revealed that the main predictors of resilience were age, years of experience, and having adequate PPE. The mental health and resilience of HCWs need to be supported to protect them from the harmful effects of mental health problems and caregiving, thereby promoting their quality of life and enhancing worldwide recovery from COVID-19 (Santarone et al., 2020). This could be achieved by tailoring appropriate interventions addressing needs and strengthening the personal resources of HCWs. In the current study, the predictors of the main variables (resilience, stress, anxiety, and depression) were varied; therefore, further studies investigating these predictors are recommended.

There are some limitations to the current study that limit generalizability. First, the use of a convenience sampling technique may lead to reporting bias. Second, data collection was done by using self-reported questionnaires (which increase the reporting bias). Additionally, the findings of the current study are limited by not measuring how many death cases the HCWs dealt with. This variable has an impact on the psychological status of HCWs; therefore, future research must highlight this.

We conclude that the mental well-being and resilience of frontline HCWs involved in treating the patients with COVID-19 are affected, mainly older HCWs. Thus, they suffer from stress, anxiety, and depression. The psychological problems among frontline HCWs should be detected as early as possible to avoid any negative consequences, whether at personal, professional, caring, or organizational levels.

Assessing risk factors of the psychological problems among HCWs, such as demographics and social characteristics, is important to apply preventive or protective measures and programs in practice settings. Additionally, these risk factors must be considered by nursing managers in practice when designing an appropriate intervention addressing the needs of HCWs to maintain their mental health. The mental health programs must be directed not only toward intervening but also toward the preparation of HCWs in practice settings before caring for COVID-19 patients. Healthcare institutions must provide a resilient environment with adequate support and resources for their staff and professions (e.g., relaxation, meditation). In Jordan, healthcare institutions must provide a healthy work environment by providing adequate PPE. Additionally, they have to provide mental health training to their HCWs to manage their stress and the stress of the patients. Free psychiatric mental health consultation must be available for HCWs in practice settings in Jordan, as they need help in maintaining confidentiality and privacy. The policymakers must tailor an appropriate policy that makes psychological service routine care as a physical one for HCWs. A national educational program about mental/psychological well-being must be adopted and applied by the Ministry of Health with the cooperation of other ministries to reduce psychological problems and their negative consequences. Future research is needed to cover the limitations of this study. Additionally, future experimental research is needed to determine the impact of providing free psychiatric consultation for the psychological problems of HCWs. In the current study, we did not measure how many death cases the HCWs dealt with. This variable might have an impact on stress, anxiety, and depression. Therefore, we recommend future research to measure it. Although the RSN showed good reliability when used to measure resilience among other HCWs (doctors, pharmacists, laboratory technicians, radiologists, or nutritionists), more studies are needed to confirm its usability, validity, and reliability among them in addition to nurses.

## Data Availability Statement

The raw data supporting the conclusions of this article will be made available by the authors, without undue reservation.

## Ethics Statement

The studies involving human participants were reviewed and approved by Philadelphia University Ethical Committee. The patients/participants provided their written informed consent to participate in this study.

## Author Contributions

Material preparation, data collection, and analysis were performed by FA, AK, OA, DY, and MK. The first draft of the manuscript was written by FA. All authors contributed to the study conception and design, commented on previous versions of the manuscript, read, and approved the final manuscript.

## Conflict of Interest

The authors declare that the research was conducted in the absence of any commercial or financial relationships that could be construed as a potential conflict of interest.

## Publisher's Note

All claims expressed in this article are solely those of the authors and do not necessarily represent those of their affiliated organizations, or those of the publisher, the editors and the reviewers. Any product that may be evaluated in this article, or claim that may be made by its manufacturer, is not guaranteed or endorsed by the publisher.

## References

[B1] Al Ammari M. Sultana K. Thomas A. Al Swaidan L. Al Harthi N. (2021). Mental health outcomes amongst health care workers during COVID-19 pandemic in Saudi Arabia. Front. Psychiatry 11:619540. 10.3389/fpsyt.2020.619540 33519559 PMC7840896

[B2] Al Mahyijari N. Badahdah A. Khamis F. (2020). The psychological impacts of COVID-19: a study of frontline physicians and nurses in the Arab world. Ir. J. Psychol. Med. 119, 1–6. 10.1017/ipm.2020.119 33109291 PMC7683819

[B3] Alnazly E. Khraisat O. M. Al-Bashaireh A. M. Bryant C. L. (2021). Anxiety, depression, stress, fear and social support during COVID-19 pandemic among Jordanian healthcare workers. PLoS ONE 16:e0247679. 10.1371/journal.pone.0247679 33711026 PMC7954309

[B4] Al-Turkait F. Ohaeri J. (2010). Dimensional and hierarchical models of depression using the Beck Depression Inventory-II in an Arab college student sample. BMC Psychiatry 10, 1–14. 10.1186/1471-244X-10-60 20670449 PMC2918548

[B5] Babore A. Lombardi L. Viceconti M. L. Pignataro S. Marino V. Crudele M.. (2020). Psychological effects of the COVID-2019 pandemic: perceived stress and coping strategies among healthcare professionals. Psychiatry Res. 293:113366. 10.1016/j.psychres.2020.113366 32798932 PMC7397939

[B6] Ben Loubir D. Serhier Z. Battas O. Agoub M. Bennani Othmani M. (2014). Evaluation of psychometric properties of the Arabic version of PSS Stress Measuring Scale in the Moroccan population. SAGE Open 4:215824401456435. 10.1177/2158244014564353

[B7] Bloom D. Cadarette D. (2019). Infectious disease threats in the twenty-first century: strengthening the global response. Front. Immunol. 10:549. 10.3389/fimmu.2019.00549 30984169 PMC6447676

[B8] Cai H. Tu B. Ma J. Chen L. Fu L. Jiang Y.. (2020). Psychological impacts and coping strategies of front-line medical staff during COVID-19 outbreak in Hunan, China. Med. Sci. Monitor 26, 1–16. 10.12659/MSM.924171 32291383 PMC7177038

[B9] Cohen S. (1988). Perceived stress in a probability sample of the United States, in The Claremont Symposium on Applied Social Psychology: The Social Psychology of Health, eds S. Spacapan and S. Oskamp (Newbury Park, CA: Sage Publications, Inc.), 31–67.

[B10] Conversano C. Marchi L. Miniati M. (2020). Psychological distress among healthcare professionals involved in the Covid-19 emergency: vulnerability and resilience factors. Clin. Neuropsychiatry 17, 94–96. 10.36131/CN20200212 PMC8629057 34908976

[B11] Dai Y. Hu G. Xiong H. Qiu H. Yuan X. (2020). Psychological impact of the coronavirus disease 2019 (COVID-19) outbreak on healthcare workers in China. medRxiv. 10, 1–22. 10.1101/2020.03.03.20030874

[B12] Derogatis L. Lipman R. Rickels K. Uhlenhuth E. Covi L. (1974). The Hopkins Symptom Checklist (HSCL): a self-report symptom inventory. Behav. Sci. 19, 1–15. 10.1002/bs.3830190102 4808738

[B13] Du J. Dong L. Wang T. Yuan C. Fu R. Zhang L.. (2020). Psychological symptoms among frontline healthcare workers during COVID-19 outbreak in Wuhan. General Hospital Psychiatry 67, 144–145. 10.1016/j.genhosppsych.2020.03.011 32381270 PMC7194721

[B14] Duan L. Zhu G. (2020). Psychological interventions for people affected by the COVID-19 epidemic. Lancet Psychiatry 7, 300–302. 10.1016/S2215-0366(20)30073-0 32085840 PMC7128328

[B15] Faul F. Erdfelder E. Lang A. Buchner A. (2013). G^*^ Power 3.1. 7: a flexible statistical power analysis program for the social, behavioral and biomedical sciences. Beh. Res. Meth. S. 39, 175–191. 10.3758/BF03193146 17695343

[B16] Guo W. P. Min Q. Gu W. W. Yu L. Xiao X. Yi W.. (2021). Prevalence of mental health problems in frontline healthcare workers after the first outbreak of COVID-19 in China: a cross-sectional study. Health Qual. Life Outcomes 19:103. 10.1186/s12955-021-01743-7 33752686 PMC7983094

[B17] Hesbacher P. T. Rickels K. Morris R. J. Newman H. Rosenfeld H. (1980). Psychiatric illness in family practice. J. Clin. Psychiatry 41, 6–10. 7351399

[B18] Jordan Armed Forces (2020). Jaf.mil.jo. Available online at: https://www.jaf.mil.jo/NewsView.aspx?NewsId=7001#.XnDtu6gzZPY [Accessed April 8, 2020].

[B19] Kalaitzaki A. Tamiolaki A. Rovithis M. (2020). The healthcare professionals amidst COVID-19 pandemic: a perspective of resilience and posttraumatic growth. Asian J. Psychiatr. 52:102172. 10.1016/j.ajp.2020.102172 32426063 PMC7227560

[B20] Kang L. Li Y. Hu S. Chen M. Yang C. Yang B.. (2020). The mental health of medical workers in Wuhan, China dealing with the 2019 novel coronavirus. Lancet Psychiatry 7:e14. 10.1016/S2215-0366(20)30047-X 32035030 PMC7129673

[B21] Kushal A. Gupta S. Mehta M. Singh M. (2018). Study of stress among health care professionals: a systemic review. Int. J. Res. Foundation Hosp. Healthc. Administr. 6, 6–11. 10.5005/jp-journals-10035-108421539165

[B22] Lai J. Ma S. Wang Y. Cai Z. Hu J. Wei N.. (2020). Factors associated with mental health outcomes among health care workers exposed to coronavirus disease 2019. JAMA Network Open 3:e203976. 10.1001/jamanetworkopen.2020.3976 32202646 PMC7090843

[B23] Li F. Luo S. Mu W. Li Y. Ye L. Zheng X.. (2021). Effects of sources of social support and resilience on the mental health of different age groups during the COVID-19 pandemic. BMC Psychiatry 21:16. 10.1186/s12888-020-03012-1 33413238 PMC7789076

[B24] Lima C. K. T. de Medeiros Carvalho P. M. Lima I. D. A. S. de Oliveira Nunes J. V. A. Saraiva J. S. de Souza R. I.. (2020). The emotional impact of coronavirus 2019-Ncov (New Coronavirus Disease). Psychiatry Res. 287:112915. 10.1016/j.psychres.2020.112915 32199182 PMC7195292

[B25] Ma Y. Rosenheck R. He H. (2020). Psychological stress among health care professionals during the 2019 novel coronavirus disease outbreak: cases from online consulting customers. Intensive Crit. Care Nurs. 61:102905. 10.1016/j.iccn.2020.102905 32712069 PMC7321034

[B26] Magnavita N. Chirico F. Garbarino S. Bragazzi N. L. Santacroce E. Zaffina S. (2021). SARS/MERS/SARS-CoV-2 outbreaks and burnout syndrome among healthcare workers: an umbrella systematic review. Int. J. Environ. Res. Public Health 18:4361. 10.3390/ijerph18084361 33924026 PMC8072681

[B27] Magnavita N. Tripepi G. Di Prinzio R. R. (2020). Symptoms in health care workers during the COVID-19 epidemic: a cross-sectional survey. Int. J. Environ. Res. Public Health 17:5218. 10.3390/ijerph17145218 32698320 PMC7400440

[B28] Mahfoud Z. Kobeissi L. Peters T. J. Araya R. Ghantous Z. Khoury B. (2013). The Arabic validation of Hopkins Symptoms Checklist-25 against MINI in a disadvantaged suburb of Beirut, Lebanon. Int. J. Educ. Psychol. Assessment 13, 17–33.

[B29] Maunder R. Hunter J. Vincent L. Bennett J. Peladeau N. Leszcz M.. (2003). The immediate psychological and occupational impact of the 2003 SARS outbreak in a teaching hospital. CMAJ 168, 1245–1251. 12743065 PMC154178

[B30] Maunder R. Lancee W. Balderson K. Bennett J. Borgundvaag B. Evans S.. (2006). Long-term psychological and occupational effects of providing hospital healthcare during SARS outbreak. Emerg. Infect. Dis. 12, 1924–1932. 10.3201/eid1212.060584 17326946 PMC3291360

[B31] McLaughlin K. Hatzenbuehler M. L. (2009). Stressful life events, anxiety sensitivity, and internalizing symptoms in adolescents. J. Abnorm. Psychol. 118, 659–669. 10.1037/a0016499 19685962 PMC2881589

[B32] Mekonen E. Shetie B. Muluneh N. (2020). The psychological impact of COVID-19 outbreak on nurses working in the northwest of Amhara regional state referral hospitals, Northwest Ethiopia. Psychol. Res. Behav. Manag. 13, 1353–1364 10.2147/PRBM.S291446 33447101 PMC7801913

[B33] Morens D. Fauci A. (2013). Emerging infectious diseases: threats to human health and global stability. PLoS Pathog. 9:e1003467. 10.1371/journal.ppat.1003467 23853589 PMC3701702

[B34] Pappa S. Ntella V. Giannakas T. Giannakoulis V. Papoutsi E. Katsaounou P. (2020). Prevalence of depression, anxiety, and insomnia among healthcare workers during the COVID-19 pandemic: a systematic review and meta-analysis. Brain Behav. Immunity 88, 901–907. 10.1016/j.bbi.2020.05.026 32437915 PMC7206431

[B35] Park J. Lee E. Park N. Choi Y. (2018). Mental health of nurses working at a government-designated hospital during a MERS-CoV outbreak: a cross-sectional study. Arch. Psychiatr. Nurs. 32, 2–6. 10.1016/j.apnu.2017.09.006 29413067 PMC7127092

[B36] Park S. Choi M. Kim S. (2019). Validation of the resilience scale for nurses (RSN). Arch. Psychiatr. Nurs. 33, 434–439. 10.1016/j.apnu.2018.12.004 31280791

[B37] Prime Ministry of Jordan (2020). رئاسة الوزراء الحكومة تعلن إجراءات جديدة للتعامل مع فيروس - كورونا تتضمن تعطيل المدارس والجامعات. Available online at: Pm.gov.jo (accessed April 8, 2020).

[B38] Robertson H. Elliott A. Burton C. Iversen L. Murchie P. Porteous T.. (2016). Resilience of primary healthcare professionals: a systematic review. Br. J. General Prac. 66, e423–e433. 10.3399/bjgp16X685261 27162208 PMC4871308

[B39] Roya (2020). First Coronavirus Case in Jordan Confirmed. En.royanews.tv. Available online at: https://en.royanews.tv/news/20134/2020-03-02 (accessed April 8, 2020).

[B40] Santarone K. McKenney M. Elkbuli A. (2020). Preserving mental health and resilience in frontline healthcare workers during COVID-19. Am. J. Emerg. Med. 38, 1515–1539. 10.1016/j.ajem.2020.04.030 32336584 PMC7156943

[B41] Selye H. (1975). Implications of stress concept. N. Y. State J. Med. 75, 2139–2145. 1059917

[B42] Shahrour G. Dardas L. A. (2020). Acute stress disorder, coping self-efficacy and subsequent psychological distress among nurses amid COVID-19. J. Nurs. Manag. 28, 1686–1695. 10.1111/jonm.13124 32767827 PMC7436502

[B43] Siyu C. Xia M. Wen W. Cui L. Yang W. Liu S.. (2020). Mental health status and coping strategy of medical workers in China during the COVID-19 outbreak. medRxiv. 10, 1–2. 10.1101/2020.02.23.20026872

[B44] Spoorthy M. S. Pratapa S. K. Mahant S. (2020). Mental health problems faced by healthcare workers due to the COVID-19 pandemic: a review. Asian J. Psychiatry 51:102119. 10.1016/j.ajp.2020.102119 32339895 PMC7175897

[B45] Vizheh M. Qorani M. Arzaghi S. M. Muhidin S. Javanmard Z. Esmaeili M. (2020). The mental health of healthcare workers in the COVID-19 pandemic: a systematic review. J. Diabetes Metab. Disord. 19, 1967–1978. 10.1007/s40200-020-00643-9 33134211 PMC7586202

[B46] Wilson W. Raj J. P. Rao S. Ghiya M. Nedungalaparambil N. M. Mundra H.. (2020). Prevalence and predictors of stress, anxiety, and depression among healthcare workers managing COVID-19 pandemic in India: a nationwide observational study. Indian J. Psychol. Med. 42, 353–358. 10.1177/0253717620933992 33398224 PMC7385435

[B47] Winokur A. Winokur D. Rickels K. Cox D. (1984). Symptoms of emotional distress in a family planning service: stability over a four-week period. Br. J. Psychiatry 144, 395–399. 10.1192/bjp.144.4.395 6722401

[B48] World Health Organization (2020). Coronavirus Disease (COVID-19) Outbreak. Who.int. Available online at: https://www.who.int/westernpacific/emergencies/covid-19 (accessed April 8, 2020).

[B49] Wu P. Fang Y. Guan Z. Fan B. Kong J. Yao Z.. (2009). The psychological impact of the SARS epidemic on hospital employees in China: exposure, risk perception, and altruistic acceptance of risk. Can. J. Psychiatry 54, 302–311. 10.1177/070674370905400504 19497162 PMC3780353

[B50] Xiang Y. Yang Y. Li W. Zhang L. Zhang Q. Cheung T.. (2020). Timely mental health care for the 2019 novel coronavirus outbreak is urgently needed. Lancet Psychiatry 7, 228–229. 10.1016/S2215-0366(20)30046-8 32032543 PMC7128153

[B51] Zandifar A. Badrfam R. (2020). Iranian mental health during the COVID-19 epidemic. Asian J. Psychiatr. 51:101990. 10.1016/j.ajp.2020.101990 32163908 PMC7128485

[B52] Zhu N. Zhang D. Wang W. Li X. Yang B. Song J. Tan W.. (2020). A novel coronavirus from patients with pneumonia in China, 2019. N. Engl. J. Med. 382, 727–733. 10.1056/NEJMoa2001017 31978945 PMC7092803

